# Breastfeeding in Turkey: practices, barriers, and cultural dynamics for global insights

**DOI:** 10.3389/fped.2025.1657654

**Published:** 2025-12-18

**Authors:** Kaan Celebier, Metin Yigit, Furkan Kalayci, Berrak Naz Kirgil, Naci Yilmaz, Ali Evrim Dogan, Orkun Tolunay

**Affiliations:** 1 Department of Pediatrics, Ankara Bilkent City Hospital, Bilkent, Ankara, Türkiye; 2 Department of Pediatrics, Yildirim Beyazit University, Ankara Bilkent City Hospital, Bilkent, Ankara, Türkiye; 3 Department of Medical, Nestle Nutrition, Istanbul, Türkiye

**Keywords:** breastfeeding practices, exclusive breastfeeding, infant nutrition, socioculturalfactors, extended family influence, breastfeeding education, formula feeding, cesareandelivery impact

## Abstract

**Objective:**

Breastfeeding is globally recognized as the cornerstone of infant nutrition, offering unparalleled benefits for maternal and child health. However, exclusive breastfeeding rates remain below international targets, influenced by sociocultural, economic, and individual factors. This study aims to investigate breastfeeding practices in Türkiye, identify influencing factors, and compare these findings with global trends to highlight both commonalities and country-specific dynamics.

**Methods:**

This cross-sectional study was conducted at Ankara Bilkent City Hospital between June and August 2024, with 392 mothers of children aged 2–5 years. Data were collected through structured surveys addressing demographics, breastfeeding practices, and formula use. Statistical analyses were performed using SPSS, with *p* < 0.05 considered significant.

**Results:**

Exclusive breastfeeding during the first six months was reported by 37.5% and was influenced by perceived milk insufficiency, socioeconomic factors, and extended family involvement. Cesarean delivery was associated with increased formula use, and lower-income families were less likely to sustain breastfeeding to 24 months. Contrary to global patterns where family support is beneficial, extended family involvement in Türkiye negatively affected exclusive breastfeeding. In multivariable models, maternal chronic illness and cesarean delivery increased the odds of formula use (OR = 1.6; 95% CI = 0.98–2.79; *p* = 0.04 and OR = 1.8; 95% CI = 1.21–2.81; *p* = 0.004). Not receiving support from extended family and normal spontaneous vaginal delivery increased the odds of exclusive breastfeeding in the first six months (OR = 1.9; 95% CI = 1.25–2.91; *p* = 0.003 and OR = 1.5; 95% CI = 1.02–2.47; *p* = 0.041). University-level education was associated with initiating complementary feeding after six months (OR = 2.1; 95% CI = 0.27–0.80; *p* = 0.006). No significant model was established for total breastfeeding duration.

**Conclusions:**

Breastfeeding practices in Turkey reflect global challenges, including maternal perceptions of insufficient milk supply and economic barriers, while also highlighting unique cultural dynamics, particularly the influence of extended family. Addressing these issues requires a dual approach: implementing universal interventions, including healthcare professional guidance and breastfeeding education, and developing culturally specific strategies to mitigate the negative impact of traditional norms on exclusive breastfeeding rates. Policymakers should prioritize reducing cesarean delivery rates and strengthening support systems for low-income mothers. Comprehensive, multifaceted interventions are essential to improve breastfeeding outcomes and align national practices with international health goals.

## Introduction

Breast milk is widely recognized as a unique biological fluid due to its exceptional properties and the comprehensive health benefits it provides for both mothers and infants (1). In addition to offering strong protection against infections, it contains probiotic compounds and live cells, which further enhance its biological value (2). Notably, its composition adapts dynamically to the infant's age and developmental stage, thereby ensuring optimal nutritional and immunological support during early life (3, 4). Given these medical advantages, the World Health Organization (WHO) and other health authorities strongly advocate exclusive breastfeeding during the first six months of an infant's life (5). In line with this recommendation, the WHO has set a global nutrition target to raise the rate of exclusive breastfeeding to 50% by the year 2025 (6).

Breastfeeding, along with complementary feeding, is recommended as the cornerstone of infant nutrition and is ideally advised to continue until the age of two (7). According to the World Health Organization (WHO), 68% of children worldwide are breastfed beyond the age of one, and increasing this rate to 80% by the year 2030 is among the organization's global targets (8). Understanding the factors that influence breastfeeding practices is crucial for guiding interventions aimed at achieving these goals. A wide range of variables—from the socio-cultural characteristics of mothers and families to the structure and efficiency of healthcare systems, and from public health and social policies to the availability of alternative feeding methods—are believed to play a significant role in determining the overall duration of breastfeeding (9).

However, despite the implementation of educational campaigns and practical interventions to raise awareness about the importance of breastfeeding, the rates of exclusive breastfeeding during the first six months postpartum have not yet met the recommended thresholds (10). Multiple variables are considered key contributors to this shortfall. Studies indicate that beyond maternal and infant health concerns or sociodemographic differences, factors such as economic status, access to breastfeeding education, maternal education level, cultural perceptions, and the mode of delivery play a decisive role in determining the success and prevalence of exclusive breastfeeding (11–13).

The decision to breastfeed is a highly personal choice, influenced by a wide range of social, cultural, and individual factors (14). In some cases, breastfeeding may be impractical, considered inappropriate, or simply insufficient, which may result in its early cessation. In such scenarios, infant formula has been introduced as an effective alternative to meet the nutritional demands of infants (15). However, dependence on infant formula—albeit unintended—may inadvertently undermine or disrupt breastfeeding patterns, with potential adverse effects on both the prevalence and sustainability of breastfeeding (16).

The primary aim of this study is to examine maternal attitudes and behaviors regarding breastfeeding, as well as the factors influencing these practices. Additionally, it seeks to identify and analyze breastfeeding practices in Turkey, with a focus on their alignment or divergence from global trends. By identifying key determinants and challenges, the study aims to generate actionable insights that promote breastfeeding and mitigate potential barriers. Furthermore, by investigating the reasons behind the substitution of breast milk with infant formula, it contributes to a deeper understanding of maternal decision-making processes. The findings are expected to inform the development of targeted policies that enhance breastfeeding support, improve maternal and infant health outcomes, and reaffirm breastfeeding as the foundation of infant nutrition.

## Materials and methods

### Study design and participants

This study was conducted at the Department of Pediatrics, Ankara Bilkent City Hospital, with a total of 412 voluntary participants. The sample included mothers of children aged 2–5 years who visited the pediatric outpatient clinics between June 2024 and August 2024, excluding those whose children had chronic illnesses. Age and chronic disease status were verified through the hospital's patient registration system to ensure the questionnaire was administered solely to mothers of children within the specified age range and without chronic conditions.

Participants were informed by the researchers during routine or outpatient visits to the pediatric clinic, and informed consent was obtained. The questionnaire was administered face-to-face to mothers who volunteered. Mothers who declined to participate, had language barriers preventing comprehension of the questionnaire, had children with chronic illnesses, or had children outside the specified age range were excluded from the study. For mothers with more than one child, only one child within the designated age group was considered, and responses did not reflect the mother's experiences with her other children. No incentives were provided for participation in the survey.

### Sample size calculation

The sample size was calculated based on a 95% confidence level, an expected prevalence of 50% (the most conservative estimate), and a margin of error of approximately 5%. According to these parameters, the minimum required sample size for the study was determined to be 384 participants. In anticipation of potential data loss or incomplete responses, a higher number of participants was targeted. Due to incomplete information, 20 questionnaires were excluded from the analysis, and a total of 392 valid questionnaires were included in the study, thus ensuring sufficient statistical power.

### Questionnaire

The questionnaire comprised 37 items and had not been previously utilized in any other study. It was developed by the authors following an extensive review of the relevant literature and tailored to the study's objectives. Ten questions assessed the socioeconomic characteristics of the participants and their families, including maternal age, education level, employment status, income level, and family structure. Six items examined the prenatal and natal characteristics of the target child. Five questions focused on the breastfeeding process, while seven addressed breastfeeding support and associated practices. The use of infant formula and the reasons for its use were investigated through four questions. Finally, five questions were devoted to evaluating the process of complementary feeding and associated preferences.

The survey did not include any trap questions. Because the questionnaire was not designed as a thematically homogeneous scale, internal consistency analysis (e.g., Cronbach's alpha) was not conducted. Items related to breastfeeding behaviors, complementary feeding, and infant formula use were evaluated based on the time elapsed since birth and the child's chronological age. The questionnaire comprised both binary (yes/no) items and multiple-choice questions allowing for the selection of more than one response. To assess its clarity and feasibility, a pilot study was conducted with 20 mothers not included in the main sample. Based on the findings, certain items were linguistically refined and instructions were clarified. The final version of the questionnaire was shaped in accordance with the feedback obtained during the pilot testing.

### Statistical analysis and ethics

Statistical analyses for the study were conducted using the Statistical Package for Social Sciences (SPSS), version 20 (IBM Corp., Armonk, NY, USA). The assumption of normal distribution for numerical variables within the study group was evaluated using the Shapiro–Wilk test. Descriptive statistics for parametric numerical data were presented as mean ± standard deviation, while non-parametric data were reported as median (minimum–maximum). Categorical data were expressed as percentages (%). The Chi-Square test was used to compare categorical variables between groups. For numerical data, the Independent Samples *T*-test was applied when the assumptions for parametric tests were met; otherwise, the Mann–Whitney U test was used. A *p*-value of less than 0.05 was considered statistically significant.

In this study, four different dependent variables—Exclusive Breastfeeding Duration for the First Six Months, Total Breastfeeding Duration, Timing of Initiation of Complementary Feeding, and Use of Formula in Infant Feeding—were analyzed using separate logistic regression models. In all models, the independent variables included Maternal Employment Status, Maternal Education Level, Monthly Household Income Level, Receiving Support from Extended Family in Childcare, Presence of Chronic Illness in the Mother, Mother's Age, Regular Antenatal Follow-up, Pregnancy Planning Status, Delivery Method, Maternal Smoking Status Before Pregnancy, Smoking During Pregnancy and Breastfeeding Period, Status of Receiving Breastfeeding Education, Source of Breastfeeding Education (Healthcare Professional), Use of Bottle in Infant Feeding, and Use of Pacifier. These variables were first evaluated by univariable logistic regression analysis, and those with *p* < 0.20 were entered into the multivariable models. The “Backward Stepwise (Likelihood Ratio)” method was applied, and the results were reported as odds ratios (OR) with 95% confidence intervals (CI) and *p*-values.

The study was conducted in accordance with the ethical principles of the Declaration of Helsinki. It was approved by the Ministry of Health of the Republic of Türkiye, as well as the Children's Hospital and the Ethics Committee of Ankara Bilkent City Hospital.

## Results

The mean age of the 392 mothers who participated in the survey was 33.88 ± 4.49 years. Of these, 256 (67.6%) reported being employed. Regarding household finances, 55 mothers (14%) indicated that their monthly income was insufficient to meet their expenses. Moreover, 243 mothers (62%) stated that they received support from extended family members in child-rearing. The demographic characteristics of the participants are detailed in Table 1.

**Table 1:** Demographic characteristics of participating mothers and their families.

Characteristic	*n* (%)/Mean ± SD
Average maternal age	33.88 ± 4.49
Average age of mother at birth	30.40 ± 4.42
Mother's education level	
Primary education	31 (7.9%)
High school	56 (14.3%)
University or Higher	305 (77.8%)
Presence of chronic disease in the mother	
Yes	80 (20.4%)
No	312 (79.6%)
Mother's Employment Status	
Employed	265 (67.6%)
Unemployed	127 (32.4%)
Father's Employment Status	
Employed	383 (97.7%)
Unemployed	9 (2.3%)
Years of Marriage	
Less than 5 Years	43 (11%)
5–10 Years	168 (42.9%)
11–15 Years	108 (27.6%)
More than 15 Years	73 (18.6%)
Income Level	
Income Less than Expenses	55 (14%)
Income Equal to Expenses	196 (50%)
Income Greater than Expenses	141 (36%)
Presence of Older Siblings for the Child	
Yes	173 (44.14%)
No	219 (55.86%)
Pregnancy planning status	
Planned pregnancy	315 (80.4%)
Unplanned pregnancy	77 (19.6%)
Regular antenatal follow-up	
Conducted	382 (97.4%)
Not conducted	10 (2.6%)
Delivery method	
Cesarean section (C/S)	252 (64.3%)
Normal spontaneous vaginal delivery (NSVD)	140 (35.7%)
Postpartum hospitalization due to illness	
Hospitalization for baby only	63 (16.1%)
Hospitalization for mother only	5 (1.3%)
Hospitalization for both mother and baby	12 (3.1%)
No hospitalization	312 (79.6%)
Support from extended family members in infant care	
Received	243 (62%)
Not received	149 (38%)
Smoking in the household	
Present	146 (37.2%)
Absent	246 (62.8%)
Maternal smoking status before pregnancy	
Non-smoker	321 (81.9%)
Smoker	71 (18.1%)
Smoking during pregnancy and breastfeeding period	
Smoked	28 (7.1%)
Did not smoke	364 (92.9%)

The breastfeeding behaviors and practices of the participating mothers were analyzed, resulting in the following findings. A total of 290 mothers (74%) initiated breastfeeding within the first hour after delivery. Regarding exclusive breastfeeding, 158 mothers (40.3%) continued between five and six months, while 147 mothers (37.5%) extended beyond six months. When asked about the total duration of breastfeeding, only 96 mothers (24.5%) reported breastfeeding for more than 24 months. A total of 230 mothers (58%) stated that they had received breastfeeding education, with 185 (47.1%) receiving it from a healthcare professional. Additionally, 178 mothers (45.4%) reported using lactation-enhancing supplements during the breastfeeding period. Breastfeeding aids, such as manual or electric breast pumps, were used by 221 mothers (56.4%). Furthermore, 244 mothers (62.2%) reported bottle-feeding their infants. The distribution of breastfeeding characteristics among participants is presented in Table 2.

**Table 2:** Breastfeeding behaviors of participating mothers.

Characteristic	*n* (%)
Timing of the first breastfeeding	
Never breastfed	2 (0.5%)
<1 h	290 (74%)
1–6 h	68 (17.3%)
6–24 h	16 (4.1%)
1–7 days	12 (3.1%)
7 days	4 (1%)
Duration of breastfeeding	
Never breastfed	2 (0.5%)
<6 months	67 (17.1%)
6–12 months	26 (6.6%)
12–18 months	63 (16%)
18–24 months	138 (35.2%)
>24 months	96 (24.5%)
Duration of exclusive breastfeeding	
Never breastfed	2 (0.5%)
<1 month	37 (9.4%)
1–5 months	48 (12.2%)
5–6 months	158 (40.3%)
>6 months	147 (37.5%)
Duration of breastfeeding for an older child	
No older child	219 (55.9%)
Older child was not breastfed	9 (2.3%)
<6 months	20 (5.1%)
6–12 months	14 (3.6%)
12–18 months	36 (9.2%)
18–24 months	57 (14.5%)
>24 months	37 (9.4%)
Status of receiving breastfeeding education	
Received	230 (58.7%)
Not received	162 (41.3%)
Source of breastfeeding education	
Not received	162 (41.3%)
Doctor	34 (8.6%)
Nurse	151 (38.5%)
Private breastfeeding coach	26 (6.7%)
Internet resources	10 (2.6%)
Extended family member	9 (2.3%)
Provision of expressed breast milk	
Provided	209 (53.3%)
Not provided	183 (46.7%)
Use of milk-enhancing products during breastfeeding period	
Used	178 (45.4%)
Not used	214 (54.6%)
Use of nutritional supplements during breastfeeding period	
Used	129 (32.9%)
Not used	263 (67.1%)
Use of breastfeeding aids	
Yes	221 (56.4%)
No	171 (43.6%)
Use of bottle in infant feeding	
Yes	244 (62.2%)
No	148 (37.8%)
Use of pacifier	
Yes	175 (44.6%)
No	217 (55.4%)

When the relationship between mothers' demographic characteristics and their breastfeeding and complementary feeding behaviors was examined, in univariate analyses variables such as maternal education, age, employment status, presence of chronic illness, pregnancy planning, prenatal follow-up, mode of delivery, and smoking history were not found to have any significant effects (*p* > 0.05 for all). These factors did not significantly influence exclusive breastfeeding during the first six months, total breastfeeding duration, or the timing of complementary feeding (*p* > 0.05 for each). Similarly, participation in breastfeeding education, receiving this education from a healthcare provider, or the regular use of pacifiers and bottles during breastfeeding had no significant impact on any of these outcomes. Notably, mothers who received support from extended family members during infant care had significantly lower rates of exclusive breastfeeding during the first six months compared to those who did not (*p* = 0.002). However, such support did not have a significant effect on the timing of introducing complementary foods or on the total duration of breastfeeding (*p* > 0.05 for both).

When examining the relationship between family income levels and the duration of breastfeeding, it was found that mothers from families whose monthly income exceeded their expenses were more likely to continue breastfeeding for up to 24 months compared to those from families with income below their expenses (*p* = 0.001). Similarly, mothers in families whose income matched their expenses also had higher rates of breastfeeding up to 24 months than those with lower income (*p* = 0.015). However, no significant difference was observed between the high-income and income-matching groups in terms of breastfeeding continuation up to 24 months (*p* > 0.05). Furthermore, family income level was not significantly associated with the initiation of complementary feeding or exclusive breastfeeding during the first six months (*p* > 0.05 for both).

Of these participants, 286 mothers (72.9%) identified increased fluid and food intake as the most effective method for boosting breast milk supply. Additionally, 77 mothers (19.8%) emphasized rest, 13 mothers (3.6%) mentioned lactation-enhancing supplements, and 14 mothers (3.7%) cited other methods. The study also examined infant formula usage and complementary feeding behaviors. A total of 236 mothers (60.2%) reported feeding their infants with formula at least once after birth, with 112 of them (28.6%) initiating formula use within the first postpartum month.

When asked about the timing of water introduction, 234 mothers (59.7%) reported giving water to their infants during the first six months of life. Additionally, 215 mothers (54.9%) stated that they introduced a non-infant formula food—not for complementary feeding purposes, but for tasting or other reasons—within the first five months. A total of 245 mothers (62.5%) initiated complementary feeding after six months, with yogurt being the most commonly chosen first complementary food. The infant formula use and complementary feeding behaviors of the participating mothers are summarized in Table 3.

**Table 3:** Formula Use and complementary feeding behaviors of participating mothers.

Characteristic	*n* (%)
Use of formula in infant feeding	
Used	236 (60.2%)
Not used	156 (39.8%)
Timing of first formula feeding	
Not used	156 (39.8%)
<1 month	112 (28.6%)
1–3 months	49 (12.5%)
3–6 months	30 (7.7%)
6–12 months	36 (9.2%)
>12 months	9 (2.3%)
Timing of first water introduction	
<1 month	20 (5.1%)
1–5 months	79 (20.1%)
5–6 months	135 (34.5%)
>6 months	158 (40.3%)
Source of support/education for complementary feeding	
Not received	146 (37.2%)
Doctor	126 (32.1%)
Nurse	19 (4.8%)
Internet resources	43 (11%)
Extended family member	56 (14.3%)
Friend	2 (0.5%)
Timing of initiation of complementary feeding	
<4 months	6 (1.5%)
4–6 months	141 (36%)
>6 months	245 (62.5%)
Provision of food during the first 5 months without initiating complementary feeding	
Yes	215 (54.9%)
No	177 (45.1%)

When 236 mothers who reported using infant formula were asked—allowing for multiple responses—about their reasons, 177 (75%) cited insufficient breast milk, 28 (11.9%) mentioned a health condition in the infant that hindered breastfeeding, 17 (7.2%) pointed to a maternal health problem, 39 (16.5%) reported that the infant refused the breast, and 9 (3.8%) indicated other reasons. Regarding factors influencing formula brand selection, 143 mothers (60.5%) cited physician recommendations, followed by pharmacist advice (24; 17.6%), nurse suggestions (37; 15.6%), recommendations from experienced friends (33; 13.9%), advertisements and promotions (15; 6.3%), product content (66; 27.9%), price (18; 7.6%), and other considerations (26; 11%).

A significant association was found between delivery mode and infant formula use (*p* = 0.005), with cesarean-delivering mothers more likely to use formula than those with vaginal deliveries. However, no significant associations were found between formula use and maternal education, age, employment status, chronic illness, planned pregnancy, prenatal follow-up, breastfeeding education, pacifier or bottle use, support from extended family, or household income (all *p* > 0.05).

According to the logistic regression analysis, several maternal and familial factors were significantly associated with infant feeding practices. Mothers with a chronic illness had a higher likelihood of formula use (OR = 1.6, 95% CI = 0.98–2.79, *p* = 0.04), and cesarean delivery was also associated with an increased likelihood of formula use (OR = 1.8, 95% CI = 1.21–2.81, *p* = 0.004). In contrast, not receiving support from extended family members in childcare increased the odds of exclusive breastfeeding for the first six months (OR = 1.9, 95% CI = 1.25–2.91, *p* = 0.003), while normal spontaneous vaginal delivery was similarly associated with higher odds of exclusive breastfeeding during the first six months (OR = 1.5, 95% CI = 1.02–2.47, *p* = 0.041). Moreover, a maternal education level of university or above was significantly related to initiating complementary feeding after six months (OR = 2.1, 95% CI = 0.27–0.80, *p* = 0.006). No significant model could be established for total breastfeeding duration. The results of our logistic regression analyses are summarized in Table 4.

**Table 4:** Determinants of breast milk and formula feeding: exclusive breastfeeding in the first Six months, total breastfeeding duration, initiation of complementary feeding after Six months, and formula use.

Model	Predictor	*P* Value	Odds Ratio	Cl Lower	Cl Upper
Formula Use	Mothers With A Chronic İllness	0.04	1.6	0.98	2.79
	Cesarean Delivery	0.004	1.8	1.21	2.81
Exclusive Breastfeeding For The First Six Months	Not Receiving Support From Extended Family Members İn Childcare	0.003	1.9	1.25	2.91
	Normal Spontaneous Vaginal Delivery	0.041	1.5	1.02	2.47
İnitiating Complementary Feeding After Six Months	Maternal Education Level Of University Or Above	0.006	2.1	0.27	0.80

CI, Confidence Interval.

Estimates are from multivariable logistic regression models; values are odds ratios with 95 percent confidence intervals.

## Discussion

Breastfeeding is the cornerstone of healthy infant nutrition (16). “Exclusive breastfeeding” refers to providing only breast milk, with no supplementation of formula or other fluids. The American Academy of Pediatrics (AAP) recommends exclusive breastfeeding for the first six months of life, followed by continued breastfeeding for at least one year or longer, depending on the mother-infant dyad. However, literature reviews show that the rate of exclusive breastfeeding declines to around 20% by six months, and many countries struggle to meet their breastfeeding targets (17, 18).

In Turkey, exclusive breastfeeding rates mirror global trends. According to the Turkish Demographic and Health Survey (TNSA), while breastfeeding is widespread, exclusive breastfeeding is not practiced at the recommended levels. The rate declined from 42% in 2008 to 30% in 2013 (19). Similarly, a study examining the reasons for the decline found that socio-demographic characteristics did not significantly affect the duration of exclusive breastfeeding (20). In our study, it was observed that participants discontinued exclusive breastfeeding within the first six months postpartum, regardless of their socio-economic status. These findings suggest that breastfeeding practices cannot be fully explained by socio-demographic factors alone, but are also shaped by awareness, knowledge, and other complex influences.

Effective management of breastfeeding during the early postpartum period is crucial for long-term success (21). Achieving this requires the guidance of qualified healthcare professionals who can assist mothers in navigating potential challenges (22). The importance of breastfeeding education during this period has been widely emphasized in the literature. For example, a study conducted in Turkey found that mothers who received breastfeeding education had higher rates of exclusive breastfeeding. The same study also reported that such education reduced maternal anxiety and positively influenced breastfeeding habits (23). Similar findings have been reported in studies from Greece and Latin American countries, further reinforcing the significance of breastfeeding education (24, 25). However, in our study, no significant difference was observed in the rates of exclusive breastfeeding during the first six months postpartum between mothers who received breastfeeding education and those who did not. Although this result may seem to contradict previous research, it can be explained by the observation that mothers in our sample often prioritized the advice of extended family members during the postpartum period. Indeed, our findings showed that mothers who received support from extended family members had lower rates of exclusive breastfeeding during the first six months. The literature frequently highlights the influential role of extended family members in childcare, particularly in shaping infant feeding practices (26–28). Although parents generally possess foundational knowledge about infant feeding, it is well documented that they do not always adhere strictly to professional recommendations (29). Practical advice from relatives and friends, along with misinformation shared through social circles, can influence parental decisions and lead to confusion (30). This confusion is especially concerning, as it may increase the risk of infants being deprived of breast milk, which is vital for their development (31, 32).

Notably, the findings of our study suggest that the influence of extended family members may be shaped by culturally specific dynamics unique to Turkey, potentially diverging from global trends. This underscores the need for locally tailored interventions to effectively address such sociocultural influences. Moreover, our study observed that the impact of extended family members extended beyond breastfeeding practices, influencing mothers' evolving informational needs and feeding decisions over time. However, this influence did not lead to significant differences in either complementary feeding behaviors or the total duration of breastfeeding. This may be attributed to mothers' increasing diversification of information sources and their tendency to seek new guidance as their infants grow and their developmental needs evolve (33). In conclusion, efforts to improve infant nutrition should not be confined to mothers alone but should encompass the entire family. Including extended family members and the wider social network in breastfeeding promotion strategies may enhance the overall effectiveness of such initiatives. Breastfeeding education and awareness campaigns are particularly valuable as cost-effective and impactful interventions. However, these efforts must go beyond individual awareness and be supported by comprehensive investments that address the broader social, cultural, and economic determinants of breastfeeding practices (34).

The benefits of breastfeeding for infant health are widely recognized and strongly supported by scientific evidence (35). Despite governmental policies and healthcare institutional efforts to promote exclusive breastfeeding, decisions regarding infant nutrition remain highly complex and are significantly shaped by individual-level determinants (34). Consequently, the use of infant formula alongside breastfeeding during the first year of life has become increasingly common. Various studies estimate the prevalence of mixed feeding (breast milk combined with formula) to range from 23% to 32%, with some reporting rates as high as 50% (36, 37). This trend is particularly prominent in high-income countries (38). Such studies underscore a global rise in infant formula use within infant nutrition. Similarly, our findings reflect this trend, showing that many mothers introduced formula in addition to breast milk during the first six months postpartum. This highlights the growing prevalence of mixed feeding practices worldwide.

When investigating the reasons for infant formula use, it was found that, in addition to maternal and infant health-related factors, maternal behaviors play a substantial role. The literature consistently highlights that one of the most influential determinants of breastfeeding behavior is the mother's perception of insufficient milk supply (39). Moreover, maternal employment has been identified in the literature as another important factor that may encourage formula use, as a demanding lifestyle can render formula feeding a more convenient and appealing alternative for some mothers (40). In our study, the most commonly reported reason for formula use was the perception of inadequate breast milk production. Other contributing factors included the infant's refusal to breastfeed, medical conditions or surgical interventions affecting the infant, prolonged maternal hospitalization, and the inability to establish successful breastfeeding during these periods.

Regarding formula brand preference, the majority of mothers reported that their choice was primarily guided by their doctor's recommendation. Additionally, recommendations from pharmacists and nurses, advice from social networks, exposure to advertising, and awareness of the product's nutritional composition also influenced brand selection. These findings suggest that both the decision to use infant formula and the choice of brand are shaped by a multifaceted set of factors. This highlights the complexity of maternal decision-making in infant nutrition and underscores the importance of considering not only individual perceptions but also external influences. Accordingly, efforts to promote breastfeeding should adopt multidimensional strategies that address both personal beliefs and the broader social and environmental context in which these decisions are made.

Cesarean delivery has been widely recognized in the literature as having potentially adverse effects on breastfeeding outcomes when compared to vaginal delivery (41). Numerous studies have demonstrated that women who undergo cesarean sections are less likely to initiate breastfeeding promptly and are more prone to experiencing delays (42, 43). These delays may compromise milk production and hinder the establishment of early mother-infant bonding (44, 45). Evidence from low- and middle-income countries further supports that cesarean delivery is associated with shorter durations of exclusive breastfeeding and a higher likelihood of infant formula use (46). Consistent with this body of research, our study also found that the mode of delivery significantly influenced infant feeding practices, particularly the use of formula.

The negative impact of cesarean delivery on breastfeeding has been attributed to multiple factors, including variations in breast milk composition and volume, difficulties initiating breastfeeding and achieving immediate skin-to-skin contact, prolonged postoperative recovery, persistent maternal pain, and delays in the development of maternal-infant attachment (47, 48). These issues are frequently cited as contributing to reduced breastfeeding success and abbreviated exclusive breastfeeding periods following cesarean birth. Collectively, these findings underscore the importance of assessing cesarean delivery not only for its medical implications but also for its broader impact on early feeding practices. This concern is especially relevant in countries with high cesarean rates. In light of these considerations, we advocate for a more judicious use of cesarean sections and the implementation of enhanced support strategies to facilitate early breastfeeding initiation among cesarean-delivered mothers.

Although low income levels did not significantly affect the duration of exclusive breastfeeding or the timing of complementary feeding initiation in our study, they were associated with a reduced overall duration of breastfeeding. This may be attributed to limited awareness of optimal breastfeeding practices among economically disadvantaged mothers, along with restricted access to breastfeeding counseling and healthcare services. Moreover, suboptimal or irregular maternal nutrition due to financial hardship may impair milk production, thereby shortening breastfeeding duration (49–51). The additional burden of domestic labor and extensive caregiving responsibilities may further hinder mothers' ability to sustain breastfeeding. These findings highlight the necessity of addressing breastfeeding support at not only the individual level but also through broader structural and policy-level interventions. Expanding free and easily accessible support services could facilitate access to accurate information and qualified care, thereby mitigating the negative effects of socioeconomic disparities. Such measures are expected to promote not only prolonged breastfeeding but also maternal psychosocial well-being and favorable attitudes toward infant health (52). In this context, policy reforms and integrated healthcare services targeting low-income mothers may serve as effective strategies for enhancing breastfeeding sustainability.

It is noteworthy that our study found no significant association between maternal employment and the total duration of breastfeeding. This finding may be attributed to the fact that employed mothers often belong to higher income brackets and may benefit from greater access to workplace support systems that facilitate breastfeeding. The literature frequently highlights the detrimental impact of low income on breastfeeding duration, linking it to the broader challenges posed by economic and social disadvantages (49). Still, it is clear that income alone cannot fully explain these dynamics. Structural and environmental factors—such as the duration of maternity leave, the availability of breastfeeding-friendly facilities at work, and the flexibility of working hours—may play a critical role in shaping breastfeeding behaviors among employed mothers. Therefore, further comprehensive studies are needed to investigate how these working conditions influence breastfeeding outcomes.

In addition, our findings showed that cesarean delivery was associated with a higher likelihood of formula use, a result that was consistent across both univariate and multivariable analyses. Similarly, receiving support from extended family members in childcare was linked to a lower prevalence of exclusive breastfeeding during the first six months, whereas the absence of such support was positively associated with maintaining exclusive breastfeeding in both analytic approaches.

Moreover, the mode of delivery emerged as a particularly striking factor: mothers who delivered by cesarean section were consistently more likely to rely on formula feeding, underscoring the potential long-term implications of delivery practices on infant nutrition. Equally noteworthy was the role of family support. Contrary to the common perception that assistance from extended family members facilitates optimal feeding, our results indicated that such support was actually associated with lower rates of exclusive breastfeeding during the first six months, whereas mothers without this support were more likely to sustain exclusive breastfeeding. These findings highlight the complex and sometimes paradoxical influence of sociocultural and perinatal factors on infant feeding behaviors.

One potential limitation of this study is its retrospective design, as data regarding breastfeeding practices were collected 2–5 years after childbirth. As such, the possibility of recall bias should be acknowledged. Moreover, the timing and duration of breastfeeding and complementary feeding education received by parents during the prenatal and postnatal periods were not examined in detail. Finally, because fathers were not included in the scope of this study, their potential influence on breastfeeding practices and infant feeding behaviors could not be assessed. In addition, although some maternal responses appeared to conflict with current infant feeding guidelines, these statements were reported without modification, as they reflect the actual behaviors and self-reported experiences of the participants.

In conclusion, our study found that the rate of exclusive breastfeeding during the first six months postpartum fell short of expected targets, influenced by a range of factors including maternal perceptions, social dynamics, and particularly the role of extended family members. The most frequently reported reasons for increased infant formula use included the perception of insufficient breast milk, advice from the social environment, and infant health issues. Notably, extended family influence appeared to contribute to a shorter overall breastfeeding duration. These findings underscore that breastfeeding practices are shaped not only by maternal attitudes and preferences but also by broader familial and environmental contexts. Accordingly, educational programs should be designed to engage not only mothers but also other family members. Awareness campaigns targeting the appropriate management of extended family influence, along with policies aimed at reducing the impact of socioeconomic disadvantage, are equally critical. Reducing reliance on infant formula should also remain a strategic priority. Ultimately, the effective promotion and support of breastfeeding requires the implementation of integrated, multilevel approaches spanning individual, societal, and policy domains.

## Data Availability

The raw data supporting the conclusions of this article will be made available by the authors, without undue reservation.
